# Including Online-Recruited Seeds: A Respondent-Driven Sample of Men Who Have Sex With Men

**DOI:** 10.2196/jmir.5258

**Published:** 2016-03-15

**Authors:** Nathan John Lachowsky, Allan Lal, Jamie I Forrest, Kiffer George Card, Zishan Cui, Paul Sereda, Ashleigh Rich, Henry Fisher Raymond, Eric A Roth, David M Moore, Robert S Hogg

**Affiliations:** ^1^British Columbia Centre for Excellence in HIV/AIDSVancouver, BCCanada; ^2^Division of AIDSFaculty of MedicineUniversity of British ColumbiaVancouver, BCCanada; ^3^School of Population and Public HealthFaculty of MedicineUniversity of British ColumbiaVancouver, BCCanada; ^4^Faculty of Health ScienceSimon Fraser UniversityBurnaby, BCCanada; ^5^Department of Epidemiology and BiostatisticsUniversity of California, San FranciscoSan Francisco, CAUnited States; ^6^San Francisco Department of Public HealthSan Francisco, CAUnited States; ^7^Department of AnthropologyFaculty of Social ScienceUniversity of VictoriaVictoria, BCCanada; ^8^Centre for Addictions Research British ColumbiaUniversity of VictoriaVictoria, BCCanada

**Keywords:** men who have sex with men, respondent driven sampling, HIV/AIDS, online recruitment, Internet

## Abstract

**Background:**

Technology has changed the way men who have sex with men (MSM) seek sex and socialize, which may impact the implementation of respondent-driven sampling (RDS) among this population. Initial participants (also known as seeds) are a critical consideration in RDS because they begin the recruitment chains. However, little information is available on how the online-recruited seeds may effect RDS implementation.

**Objective:**

The objectives of this study were to compare (1) online-recruited versus offline-recruited seeds and (2) subsequent recruitment chains of online-recruited versus offline-recruited seeds.

**Methods:**

Between 2012 and 2014, we recruited MSM using RDS in Vancouver, Canada. RDS weights were used with logistic regression to address each objective.

**Results:**

A total of 119 seeds were used, 85 of whom were online-recruited seeds, to recruit an additional 600 MSM. Compared with offline-recruited seeds, online-recruited seeds were less likely to be HIV-positive (OR 0.34, 95% CI 0.13-0.88), to have attended a gay community group (AOR 0.33, 95% CI 0.12-0.90), and to feel gay community involvement was “very important” (AOR 0.16, 95% CI 0.03-0.93). Online-recruited seeds were more likely to ask a sexual partner’s HIV status always versus <50% of the time (AOR 5.21, 95% CI 1.17-23.23), to have watched the Pride parade (AOR 6.30, 95% CI 1.69-23.45), and to have sought sex online (AOR 4.29, 95% CI 1.53-12-12.05). Further, compared with recruitment chains started by offline-recruited seeds, recruits from chains started by online-recruited seeds (283/600, 47.2%) were less likely to be HIV-positive (AOR 0.25, 95% CI 0.16-0.40), to report “versatile” versus “bottom” sexual position preference (AOR 0.56, 95% CI 0.35-0.88), and to be in a relationship lasting >1 year (AOR 1.65, 95% CI 1.06-2.56). Recruits of online seeds were more likely to be out as gay for longer (eg, 11-21 vs 1-4 years, AOR 2.22, 95% CI 1.27-3.88) and have fewer Facebook friends (eg, 201-500 vs >500, AOR 1.69, 95% CI 1.02-2.80).

**Conclusions:**

Online-recruited seeds were more prevalent, recruited fewer participants, but were different from those recruited offline. This may therefore help create a more diverse overall sample. Our work has shown the value of geosocial networking apps for aiding RDS recruitment efforts, especially when faced with slow participation uptake by other means. Understanding the degree to which networks interact will be an important next step in confirming the efficacy of online RDS recruitment strategies.

## Introduction

Respondent driven sampling (RDS), a form of peer referral-based sampling, has become a popular strategy to recruit “hidden” or “marginalized populations” [1,2]. This rise in use has led some researchers to critically assess both theoretical and operational components of the methodology. RDS implementation resembles snowball sampling with several critical caveats [3-6]. Initial participants are purposefully recruited to be “seeds” as long as they fit the study’s eligibility criteria. After completing the study procedures, seeds are offered a limited number of vouchers to recruit their peers to participate. When vouchers are redeemed, eligible participants also complete the same study procedures and are asked to recruit their peers, and this continues until recruitment goals are met. Using specially formulated statistical programs, sampling weights are developed and applied to estimate population parameters. For the purpose of RDS, effective seeds generate large recruitment chains and samples, which has been shown to be associated with motivation and a commitment to the research goals [7].

While many researchers have pointed to the issues around the statistical accuracy and precision of estimates generated from this sampling methodology [2,8,9], few have discussed the practice, innovation, and implementation of RDS. There is growing interest in online-based methods to address the prevalence of Internet use among men who have sex with men (MSM) and acknowledge the growth of online social and sexual networking [10,11]. Innovation around RDS can be helpful in mitigating some of the challenges of implementing the methodology, notably slow recruitment rates [5,12].

The Internet and the rise of online communities of gay and bisexual men remains an underutilized tool for population health researchers. In only a few decades, there has been a rapid uptake of the Internet among gay and bisexual men to facilitate new connections [13,14]. As many as 90% of gay and bisexual men have reported using the Internet for information seeking and socializing [14]. Other reports estimate that between 40% and 97% use the Internet to seek sex [15,16]. With a high prevalence of Internet usage among this population, new connections and networks are formed online now more than ever before [13,17]. As such, the use of traditional venue-based and time-location sampling may reach different segments of the study population at different times [18].

Prior formative research related to implementing RDS identified the importance of having diverse seeds to generate a sample with a greater ability to penetrate harder-to-reach subgroups of the population [1]. Furthermore, previous work has shown that diversity in seed selection methods can produce varying samples but more importantly differing recruitment rates [18,19]. Given increased utilization of specific online virtual spaces among MSM in Vancouver, Canada [20], we supplemented our RDS strategy by recruiting seeds online through a popular geolocation social networking mobile phone app, Grindr, used by MSM to seek sex with other men. The objectives of this analysis were to determine whether there were any meaningful differences in the seeds that we recruited online compared with those recruited through other community venues, and whether participants recruited in network chains that started with an online-recruited seed differed from those participants recruited from other seeds.

## Methods

### Recruitment

The Momentum Health Study is a longitudinal cohort of gay, bisexual, and other men who have sex with men in Metro Vancouver, British Columbia, Canada, recruited by RDS. The study aims to report on the effect of expanded access to antiretroviral therapy for the treatment of human immunodeficiency virus (HIV) and is the first study to use RDS in the greater Vancouver area to obtain a diverse sample of MSM. Prior to recruitment, we conducted systematic qualitative formative research to map the network structure of MSM in Vancouver and the surrounding area [20].

In February 2012, we began recruitment using 10 initial seeds selected from various community agency partners. Each seed was given three RDS coupons (electronic or laminated card or combination). Initial seeds were instructed to distribute RDS vouchers to other MSM in their social or sexual networks who could then redeem the card to participate in the study. Like many previous studies, we initially distributed three RDS coupons to each participant, but after encountering substantially slow recruitment rates in the first 6 months, we increased the number of RDS coupons from three to six. However, we observed no significant difference in rate of recruitment. Since seeds can be purposively selected [21,22], we decided to utilize a popular networking app for gay and bisexual men (ie, Grindr) to recruit additional seeds. Advertisements in the form of pop-up messages were sent to users within a 25 km radius to downtown Vancouver. The message contained a link to information about the study and an opportunity to leave a message for study staff to contact them by email or phone. In total, study staff sent out nine broadcast messages during the recruitment period.

A CAN $50 honorarium or equivalent entries into a draw for electronics or travel gift cards (CAN $10/entry) was provided to participants who completed the study protocol. A CAN $10 honorarium was provided for each additional recruit who successfully completed the study protocol (to a maximum of 6 recruits/CAN $60).

### Independent Variables

In comparing the characteristics and recruitment efficacy of online-recruited seeds with other seeds, we classified each seed as either “online” if they were recruited through the sociosexual networking app advertisement, or as “offline” if they were selected as part of the initial study protocol. Among all study seeds, we compared “online” with “offline” seeds. We also compared the subsequent recruitment chains of “online” with “offline” seeds to compare the sample derived from online versus offline seeds.

### Dependent Variables

We investigated a number of sociodemographic, community, and sexual behavioral factors thought to be associated with recruitment RDS dynamics. Sociodemographic variables included age (continuous in years), sexual identity (gay vs all other identity options collapsed: bisexual, queer, questioning, straight, etc), race/ethnicity (white vs all other identity options collapsed: Asian, Aboriginal, Latino, Black, other), birthplace (Canada vs any other country), formal educational attainment (any post-secondary education versus none), current student (or not), currently employed (or not), and annual income (<CAN $30,000 vs ≥$30,000). Living in a house, apartment, or student residence were collapsed together and considered being stably housed versus those who reported being homeless or in temporary housing. HIV status was determined using a point-of-care rapid HIV test administered at the time of the study visit (HIV-positive vs HIV-negative).

Community factors included questions about the gender of those they socialize most with (mostly or only men vs mostly or only women), how many years they consider themselves to have been “out” categorized into quartiles for those who were gay-identified (1-4, 5-10, 11-21, >21, or bisexual-identified, or not out), participation on a gay sports team in the past 6 month (yes vs no), having attended a group or club for gay men in the past 6 months (yes vs no), having attended the Pride parade in the past year (as a volunteer or in the parade vs as a spectator vs not at all), and having used a smartphone/mobile app to seek sex with another man in the past 6 months (yes vs no). Participants were asked to indicate “how important is it to be connected to and involved in the gay community” on a 4-point ordinal scale (not at all, not very or somewhat important, very important). Finally, participants were asked to indicate how many Facebook friends they had on a continuous measure, which was used as a proxy for (online) social network (0-30, 31-200, 201-500, >500).

Sexual behavioral factors included any sex with any females in the past 2 years (yes vs no), male anal sex position preference (bottom, versatile, top, or prefer no anal), marital or common law status as indicated by living together for at least 1 year (not married or common law, married or common law, no regular partner), number of male anal sex partners in the past 6 months measured continuously but categorized into quartiles (0-1, 2-3, 4-7, >7), if they had received any income for sex in the past 6 months (or not), how often they ask their partner’s HIV status (less than half the time, more than half the time, or always), whether they had ever been tested for HIV (or not), and whether they have engaged in any “risk sex” defined as any condomless anal intercourse with a partner of opposite or unknown HIV status in the past 6 months.

### Statistical Analysis

For all analyses, including the descriptive statistics, RDS-weighted adjustments were generated using RDSAT version 7.1.46, taking into account the network size and homophily. The RDS-derived weights were then imported into SAS (version 9.3) for inclusion in the bivariate and multivariable logistic regression analyses. Two final multivariable logistic regression models were built using backward selection criteria that minimized the Aikaike Information Criterion. The first model compared differences between online-recruited and offline-recruited seeds. The second model compared differences between the samples recruited from online-recruited seeds and offline-recruited seeds. All tests were two-sided and performed with alpha set at the .05 significance level.

Ethical approval for this study was been granted by the research ethics boards of Simon Fraser University, University of British Columbia Providence Healthcare Research Institute, and the University of Victoria.

## Results

A total of 119 seeds were used; 85 were recruited online (71.4%). These 119 seeds recruited a subsequent 600 participants for a total sample size of 719 MSM. Most of these seeds cited the following reasons for wanting to participate: help the community (40/119, 33.6%), had an interest in sexual health and HIV (35/119, 29.4%), and interested in gay men’s issues (23/119, 19.3%). Online-recruited seeds had significantly smaller networks than other seeds (median 10 vs 15, *P*=.006).

Table 1 provides descriptive statistics and bivariate associations with being an online-recruited seed compared with being an in-person recruited seed. Online recruited seeds were less likely to be HIV-positive (OR 0.34, 95% CI 0.13-0.88), report attending gay-community meetings in the past 6 months (OR 0.27, 95% CI 0.12-0.63), and report that being involved in the gay community was very important vs not important (OR 0.11, 95% CI 0.02-0.54), but were more likely to attend a Pride parade in the past 12 months as a spectator (OR 2.84, 95% CI 1.05-7.69), use apps to seek sex in the past 6 months (OR 3.41, 95% CI 1.49-7.82), and have 31-200 Facebook friends compared to over 500 (OR 3.47, 95% CI 1.07-11.19).

**Table 1:** Descriptive statistics of and bivariate associations with being an online-recruited seed compared with in-person-recruited seed.

			Offline seeds (n=34)		Online seeds (n=85)		OR	95% CI
			n	% (of row)	n	% (of row)		
**Demographic factors**								
	Age: mean (Q1,Q3)		28.5	(24,38)	31	(25,45)	1.020	0.985-1.056
	**Gay-identified**							
		Yes	31	28.2	79	71.8	Ref	
		No	3	33.3	6	66.7	0.785	0.185-3.335
	**White race/ethnicity**							
		Yes	28	82.4	63	69.2	Ref	
		No	6	17.7	22	78.6	1.629	0.595-4.457
	**Born in Canada**							
		Yes	28	32.2	59	67.8	Ref	
		No	6	18.8	26	81.3	2.056	0.760-5.563
	**Formal education**							
		High school or less	4	23.5	13	76.5	Ref	
		Any post-secondary training	30	30.0	70	70.0	0.718	0.216-2.383
	**Current student**							
		No	25	29.4	60	70.6	Ref	
		Yes	9	26.5	25	73.5	1.157	0.474-2.828
	**Annual income, CAD**							
		<$30,000	21	30.4	48	69.6	Ref	
		≥$30,000	13	26.0	37	74.0	1.245	0.552-2.810
	**Currently employed**							
		No	10	33.3	20	66.7	Ref	
		Yes	24	27.0	65	73.0	1.354	0.555-3.304
	**Currently stably housed**							
		Yes	33	29.5	79	70.5	Ref	
		No	1	14.3	6	85.7	2.506	0.290-21.637
	**HIV test result**							
		Negative	23	24.0	73	76.0	Ref	
		Positive	11	47.8	12	52.2	0.344^a^	0.134-0.882
**Community factors**								
	**Only/Mostly socialize with men**							
		Yes	21	32.3	44	67.7	Ref	
		No	13	24.1	41	75.9	1.505	0.668-3.390
	**Play on gay sports team, past 6 months**							
		No	25	25.0	75	75.0	Ref	
		Yes	9	47.4	10	52.6	0.370	0.135-1.015
	**Attend gay-specific groups, past 6 months**							
		No	13	18.1	59	81.9	Ref	
		Yes	21	44.7	26	55.3	0.273^a^	0.119-0.627
	**Pride parade participation, past 12 months**							
		No	12	34.3	23	65.7	Ref	
		Yes, spectator	9	15.5	49	84.5	2.841^a^	1.049-7.692
		Yes, in or volunteer	13	50.0	13	50.0	0.522	0.185-1.474
	**Use apps to seek sex, past 6 months**							
		No	19	45.2	23	54.8	Ref	
		Yes	15	19.5	62	80.5	3.414^a^	1.490-7.823
	**Importance of being connected to and involved in gay community**							
		Not important	2	8.0	23	92.0	Ref	
		Somehow important	14	26.4	39	73.6	0.242	0.050-1.163
		Very important	18	43.9	23	56.1	0.111^a^	0.023-0.535
	**# of Facebook friends**							
		>500	14	40.0	21	60.0	Ref	
		201-500	10	29.4	24	70.6	1.600	0.588-4.353
		31-200	5	16.1	26	83.9	3.467^a^	1.074-11.191
		0-30	5	26.3	14	73.7	1.867	0.548-6.353
**Sex & HIV factors**								
	**Any sex with females**							
		No	32	28.6	80	71.4	Ref	
		Yes	2	28.6	5	71.4	1.000	0.184-5.421
	**Male anal sex position preference**							
		Bottom	13	32.5	27	67.5	Ref	
		Versatile	8	29.6	19	70.4	1.144	0.397-3.295
		Top	12	25.0	36	75.0	1.444	0.570-3.660
		No anal	1	25.0	3	75.0	1.444	0.137-15.266
	**Common law (lived together 1 year)**							
		No	8	42.1	11	57.9	Ref	
		Common Law/Married	6	30.0	14	70.0	1.697	0.453-6.358
		No regular partner	20	25.0	60	75.0	2.182	0.770-6.186
	**# Male anal sex partners, past 6 months**							
		0-1	11	35.5	20	64.5	Ref	
		2-3	9	32.1	19	67.9	1.161	0.394-3.425
		4-7	6	20.0	24	80.0	2.200	0.691-7.006
		>7	8	26.7	22	73.3	1.513	0.507-4.515
	**Any income from sex work, past 6 months**							
		No	32	28.3	81	71.7	Ref	
		Yes	2	33.3	4	66.7	0.790	0.138-4.529
	**Asks partner’s HIV status**							
		<50% of time	16	34.8	30	65.2	Ref	
		>50% of time	14	31.1	31	68.9	1.181	0.492-2.834
		100% of time	4	14.3	24	85.7	3.200	0.945-10.839
	**HIV test, ever**							
		No	2	40.0	3	60.0	Ref	
		Yes	32	28.1	82	71.9	1.709	0.273-10.707
	**Any risky sex** ^b^ **, past 6 months**							
		No	22	26.5	61	73.5	Ref	
		Yes	12	34.3	23	65.7	0.691	0.295-1.619

^a^Indicates statistical significance at *P*<.05.

^b^Any condomless anal intercourse with a partner of opposite or unknown HIV status.

Table 2 shows the results of the multivariable model identifying factors associated with being an online-recruited seed. In these analyses, online recruited seeds were less likely to feel it was “very important” to be involved in the gay community versus finding it not important (AOR 0.16, 95% CI 0.03-0.93), to have attended gay specific groups in the past 6 months (AOR 0.33, 95% CI 0.12-0.90), and more likely to have been a spectator at the Pride parade in the prior 12 months versus not (AOR 6.30, 95% CI 1.69-23.45), to ask a sexual partners HIV status 100% of the time versus less than 50% of the time (AOR 5.21, 95% CI 1.17-23.23), and to have used an online application to seek sex in the past 6 months than not (AOR 4.29, 95% CI 1.53-12.05).

Although online-recruited seeds comprised 71.4% of all seeds, their subsequent recruitment chains generated only 47.2% of the remaining sample (283/600). Table 3 shows the descriptive statistics and bivariate associates with being recruited from an online-recruited seed chain compared with an in-person-recruited seed’s chain.

**Table 2:** Multivariable analyses of factors associated with being an online-recruited seed compared with in-person-recruited seed.

		AOR	95% CI
**Attend gay-specific groups, past 6 months**			
	No	Ref	
	Yes	0.331^a^	0.122-0.900
**Pride parade participation, past 12 months**			
	No	Ref	
	Yes, spectator	6.303^a^	1.694-23.454
	Yes, in or volunteer	1.192	0.325-4.369
**Use apps to seek sex, past 6 months**			
	No	Ref	
	Yes	4.293^a^	1.530-12.048
**Importance of being connected to and involved in gay community**			
	Not important	Ref	
	Somehow important	0.380	0.072-2.016
	Very important	0.163^a^	0.029-0.929
**Asks partner’s HIV status**			
	<50% of time	Ref	
	>50% of time	1.032	0.343-3.104
	100% of time	5.211^a^	1.169-23.232

^a^Indicates statistical significance at *P*<.05.

**Table 3:** Descriptive statistics of and bivariate associations with being recruited in a chain started from an online seed compared with a recruitment chain started with an in-person-recruited seed.

			Offline seed’s recruitment chain (n=317)		Online seed’s recruitment chain (n=283)		OR	95% CI
			n	% (of row)	n	% (of row)		
**Demographic factors**								
	Age: mean (Q1,Q3)		40	(28,49)	31	(25,41)	0.962^a^	0.949-0.976
	**Gay-identified**							
		Yes	265	52.8	237	47.2	Ref	
		No	52	53.1	46	46.9	0.989	0.641-1.526
	**White race/ethnicity**							
		Yes	240	53.6	208	46.4	Ref	
		No	77	50.7	75	49.3	1.124	0.778-1.624
	**Born in Canada**							
		Yes	258	54.9	212	45.1	Ref	
		No	59	45.4	71	54.6	1.465	0.991-2.163
	**Formal education**							
		High school or less	88	58.3	63	41.7	Ref	
		Any post-secondary training	224	51.3	213	48.7	1.328	0.914-1.930
	**Current student**							
		No	268	55.5	215	44.5	Ref	
		Yes	49	42.2	67	57.8	1.704^a^	1.131-2.568
	**Annual income, CAD**							
		<30,000	216	55.7	172	44.3	Ref	
		≥$30,000	101	47.6	111	52.4	1.380	0.986-1.931
	**Currently employed**							
		No	144	61.5	90	38.5	Ref	
		Yes	173	47.3	193	52.7	1.785^a^	1.278-2.492
	**Currently stably housed**							
		Yes	262	51.5	247	48.5	Ref	
		No	55	60.4	36	39.6	0.694	0.441-1.094
	**HIV test result**							
		Negative	182	42.9	242	57.1	Ref	
		Positive	135	76.7	41	23.3	0.228^a^	0.153-0.340
**Community factors**								
	**Only/mostly socialize with men**							
		Yes	187	55.5	150	44.5	Ref	
		No	130	49.4	133	50.6	1.275	0.923-1.762
	**Years out**							
		1-4	52	52.0	48	48.0	Ref	
		5-10	56	45.5	67	54.5	1.296	0.764-2.200
		11-21	61	40.7	89	59.3	1.581	0.949-2.632
		>21	107	71.8	42	28.2	0.425^a^	0.250-0.723
		Bisexual	35	55.6	28	44.4	0.867	0.460-1.632
		Not out	6	40.0	9	60.0	1.625	0.538-4.906
	**Play on gay sports team, past 6 months**							
		No	287	53.4	250	46.6	Ref	
		Yes	30	47.6	33	52.4	1.263	0.749-2.131
	**Attend gay-specific groups, past 6 months**							
		No	193	52.7	173	47.3	Ref	
		Yes	124	53.0	110	47.0	0.990	0.712-1.375
	**Pride parade participation, past 12 months**							
		No	125	54.1	106	45.9	Ref	
		Yes, spectator	159	54.1	135	45.9	1.001	0.709-1.415
		Yes, in or volunteer	33	44.0	42	56.0	1.501	0.888-2.535
	**Use apps to seek sex, past 6 months**							
		No	177	59.6	120	40.4	Ref	
		Yes	140	46.2	163	53.8	1.717^a^	1.243-2.374
	**Importance of being connected to and involved in gay community**							
		Not important	62	45.3	75	54.7	Ref	
		Somehow important	168	54.7	139	45.3	0.684	0.456-1.025
		Very important	87	55.8	69	44.2	0.656	0.413-1.040
	**# of Facebook friends**							
		>500	77	51.3	73	48.7	Ref	
		201-500	53	40.2	79	59.8	1.572	0.980-2.523
		31-200	73	49.0	76	51.0	1.098	0.698-1.728
		0-30	114	67.5	55	32.5	0.509^a^	0.323-0.801
**Sex & HIV factors**								
	**Any sex with females**							
		No	266	52.7	239	47.3	Ref	
		Yes	51	53.7	44	46.3	0.960	0.619-1.490
	**Male anal sex position preference**							
		Bottom	96	47.8	105	52.2	Ref	
		Versatile	98	59.0	68	41.0	0.634^a^	0.419-0.961
		Top	107	52.2	98	47.8	0.837	0.567-1.236
		No anal	16	57.1	12	42.9	0.686	0.309-1.523
	**Common law (lived together 1 year)**							
		No	56	43.4	73	56.6	Ref	
		Common law/Married	54	51.4	51	48.6	0.725	0.432-1.215
		No regular partner	207	56.6	159	43.4	0.589^a^	0.393-0.883
	**# Male anal sex partners, past 6 months**							
		0-1	90	54.5	75	45.5	Ref	
		2-3	71	46.1	83	53.9	1.403	0.903-2.180
		4-7	68	50.7	66	49.3	1.165	0.738-1.839
		>7	87	59.6	59	40.4	0.814	0.519-1.277
	**Any income from sex work, past 6 months**							
		No	296	52.9	264	47.1	Ref	
		Yes	21	52.5	19	47.5	1.014	0.534-1.928
	**Asks partner’s HIV status**							
		<50% of time	137	51.9	127	48.1	Ref	
		>50% of time	98	49.0	102	51.0	1.123	0.777-1.622
		100% of time	82	60.3	54	39.7	0.710	0.467-1.081
	**HIV test, ever**							
		No	13	38.2	21	61.8	Ref	
		Yes	304	53.7	262	46.3	0.534	0.262-1.086
	**Any risky sex** ^b^ **, past 6 months**							
		No	180	50.3	178	49.7	Ref	
		Yes	129	56.8	98	43.2	0.768	0.550-1.073

^a^Indicates statistical significance at *P*<.05.

^b^Any condomless anal intercourse with a partner of opposite or unknown HIV status.

These aforementioned associations show that participants recruited from an online-recruited seeds’ chains were less likely to be older (OR 0.96, 95% CI 0.95-0.98), HIV-positive (OR 0.23, 95% CI 0.15-0.34), be versatile than a bottom with regards to sexual position preferences (OR 0.63, 95% CI 0.42-0.96), be in a relationship lasting more than 1 year versus being single (OR 0.59, 95% CI 0.39-0.88), to have between 0 and 30 Facebook friends compared with having more than 500 Facebook friends (OR 0.51, 95% CI 0.32-0.80), been “out” for over 22 years compared with between 1 and 4 years (OR 0.43, 95% CI 0.25-0.72), but were more likely to be currently employed (OR 1.79, 95% CI 1.28-2.49), be a current student (OR 1.70; 95% CI 1.13-2.57), and use apps to seek sex in the past 6 months (OR 1.72, 95% CI 1.24-2.37).

Table 4 reports the results of the multivariable analysis of factors associated with being recruited in a network chain that originated from an online-recruited seed. These results also showed that participants who were recruited from online-recruited seeds’ chains were less likely to be HIV-positive (AOR 0.25, 95% CI 0.16-0.40), be versatile as their sexual position preference (AOR 0.56, 95% CI 0.35-0.88), be in a relationship lasting ˃1 year versus being single (AOR 1.65, 95% CI 1.06-2.56). However, these participants were more likely to be have been “out” for 11-21 versus 1-4 years (AOR 2.22, 95% CI 1.27-3.88) and to have between 201-500 Facebook friends versus over 500 (AOR 1.69, 95% CI 1.02-2.80).

**Table 4:** Multivariable analyses of factors associated with being recruited in a chain started from an online seed compared with a recruitment chain started with an in-person-recruited seed.

		AOR	95% CI
**HIV test result**			
	Negative	Ref	
	Positive	0.250^a^	0.158-0.396
**Years out**			
	1-4	Ref	
	5-10	1.300	0.749-2.256
	11-21	2.215^a^	1.266-3.877
	22	0.929	0.500-1.724
	Bisexual-identified	1.492	0.738-3.015
	Not out	2.182	0.662-7.192
**# of Facebook friends**			
	>500	Ref	
	201-500	1.689^a^	1.020-2.796
	31-200	1.430	0.864-2.367
	0-30	0.910	0.536-1.544
**Male anal sex position preference**			
	Bottom	Ref	
	Versatile	0.556^a^	0.352-0.877
	Top	0.708	0.459-1.091
	No anal	0.552	0.226-1.346
**Common law (lived together 1 year)**			
	No	Ref	
	Common law/Married	0.711	0.401-1.259
	No regular partner	0.605^a^	0.390-0.939

^a^Indicates statistical significance at *P*<.05.

## Discussion

### Principal Results

In this RDS study of MSM in Vancouver, a total of 119 seeds were used to recruit a subsequent 600 participants. Nearly three-quarters of the seeds used were recruited online, which was meant to address limitations of slow recruitment rates [5,12], but the online seeds recruited less than half of the subsequent sample. As such, these online-recruited seeds were not able to generate as large recruitment chains as those seeds recruited more traditionally through community agencies, which may be a result of different motivations and social networks [7]. Our findings demonstrated that online-recruited seeds who favored using hook-up apps to seek sex were less likely to attend gay-specific groups and felt less importance with respect to participating in the larger gay community. These findings are consistent with previous work indicating that gay men recruited online tend to be different with regard to behavioral and even personal characteristics in comparison with those recruited at other venues [23-25].

Online-recruited seeds and their subsequent recruits were more likely to be HIV-negative, to use apps to seek sex with other men, and to report fewer Facebook friends, a proxy for (online) social network size. We also found that men recruited online were more likely to inquire about a sex partner’s HIV status before sex. This suggests that men who participate in online sex seeking may have better communication opportunities to inform and/or interest to adopt more seroadaptive behaviors (ie, sex that uses knowledge of HIV status to inform practices that reduce the risk of HIV transmission) into their sexual practices [26]. This includes serosorting and strategic positioning decisions, which can help reduce HIV transmission. In San Francisco, researchers reported that seroadaptive behaviors are common and have increased in the last decade [27]. This may reflect the changing ways that gay and bisexual men seek and integrate sexual health knowledge that is related to the rise in seeking sex in online spaces.

### Comparison With Prior Work

Importantly for the consideration of implementing RDS among urban gay and bisexual men, we found that neither online-recruited seeds nor their recruits were significantly different than the comparator with respect to demographic factors. This is in contrast to previous RDS work that also implemented online or innovative recruitment protocols. Previous studies using RDS recruitment combined with online or self-selected innovations found significant differences with regards to ethnicity, economic status, and education [19,28,29]. This key difference in findings may suggest that online and offline networks are increasingly overlapped as greater numbers of MSM now socialize and seek sex online. It may also reflect regional and national differences in community (both physical and virtual) participation and sex seeking norms.

### Limitations

The novel use of a sociosexual mobile app to recruit seeds for RDS allowed us to reach a wider non–location-based audience of the target population interested in participating in a sexual health research study. However, it is important to note that these findings do not compare the characteristics of MSM who self-selected into the study and those who saw our mobile app advertisement but did not respond. Therefore, we cannot be certain whether there are differences among the general online and offline populations and how these differences potentially introduce self-selection biases into our recruitment efforts. RDS employs peer recruitment that requires participants to know someone else’s eligibility status for the study and to disclose their own sexual minority behavior, which also limits generalizability. Our results may also be limited by the fact that online seeds were recruited through an app used for sex seeking, while offline seeds were recruited through community partners whose mission and purpose go far beyond sex seeking. Therefore, it is difficult to assess what differences are attributable to the nature of the recruitment site versus the actual medium of recruitment.

### Conclusions

In conclusion, our work has shown the value of geosocial networking apps for aiding RDS recruitment efforts, especially when faced with slow participation uptake by other means. Understanding the degree to which networks interact will be an important next step in confirming the efficacy of online RDS recruitment strategies. Our findings highlighted some differences between online and offline seeds, indicating that, in the current sociosexual milieu, using both online- and traditionally recruited seeds may provide the most diverse sample of MSM in urban areas.

## Abbreviations

HIV: human immunodeficiency virus
MSM: men who have sex with men
RDS: respondent-driven sampling
